# A Case Report of Primary Intraosseous Carcinoma of the Mandible Initially Diagnosed as Mandibular Osteomyelitis

**DOI:** 10.7759/cureus.78892

**Published:** 2025-02-12

**Authors:** Kei-ichiro Miura, Naoki Katase, Misa Sumi, Masahiro Umeda, Tomohiro Yamada

**Affiliations:** 1 Department of Oral and Maxillofacial Surgery, Nagasaki University Graduate School of Biomedical Sciences, Nagasaki, JPN; 2 Department of Oral Pathology, Nagasaki University, Nagasaki, JPN; 3 Department of Radiology and Biomedical Informatics, Nagasaki University Graduate School of Biomedical Sciences, Nagasaki, JPN; 4 Department of Oral and Maxillofacial Surgery, Nagasaki University, Nagasaki, JPN

**Keywords:** bone resorption, early diagnosis, mandibular osteomyelitis, odontogenic tumor, primary intraosseous carcinoma, squamous cell carcinoma, surgical treatment, tooth extraction biopsy

## Abstract

Primary intraosseous carcinoma (PIOC) is a rare malignant tumor of the jaw, often diagnosed as odontogenic cysts or osteomyelitis. We report a case of a 53-year-old woman who was initially diagnosed with mandibular osteomyelitis resulting from pericoronitis of the left lower third molar. The patient underwent tooth extraction under general anesthesia, and the granulation tissue from the extraction socket was sent for histopathological evaluation, revealing moderately differentiated squamous cell carcinoma. Postoperative imaging revealed significant bone resorption, invasion of the mandibular canal, and proximity to a left submandibular lymph node. Based on these findings, the patient was diagnosed with PIOC and subsequently underwent left-sided neck dissection, hemi-mandibulectomy, and reconstructive surgery. Histopathological examination confirmed the diagnosis of PIOC, with no evidence of metastatic lymph nodes. The patient has remained disease-free for seven years and four months postoperatively. PIOC is believed to originate from odontogenic cysts and is often detected at advanced stages due to its intraosseous location. Diagnosis is based on the absence of continuity with the oral mucosa, absence of metastatic oral tumor, and presence of squamous cell carcinoma. Early diagnosis and treatment are crucial for improving patient prognosis and quality of life. This case underscores the importance of considering malignancy in cases of destructive bone resorption and highlights the importance of timely biopsy in preventing delayed treatment.

## Introduction

Primary intraosseous carcinoma (PIOC) is a rare malignant tumor, and its essential diagnostic criteria include destructive central jaw lesions, absence of communication with the surface mucosa or antrum, and exclusion of metastatic disease [1]. A previous study has reported that PIOC is predominantly diagnosed in male patients (69.3%), with a mean age of 57.3 years (range: 5-89 years). Anatomically, PIOC most commonly affects the mandible, with a mandible-to-maxilla ratio of 7:1, and is primarily located in the posterior region of the mandible. Radiographically, 31.1% of cases have been reported to exhibit irregular lesions with ill-defined margins, and cortical bone destruction has been identified in 33.5% of cases [2]. According to the fifth edition of the World Health Organization classification of head and neck tumors, it is classified as PIOC, not otherwise specified (NOS) [3]. PIOC, NOS is usually squamous carcinoma with variable differentiation, most commonly exhibiting moderate differentiation. A recent systematic review reported that the majority of cases arise from odontogenic cysts, with residual and radicular cysts being the most common origins, followed by dentigerous and odontogenic keratocysts [2,4]. In the 2022 WHO classification, it is categorized as a "malignant odontogenic tumor" within the broader group of "odontogenic tumors" [1]. The strict diagnosis of PIOC in the oral cavity is challenging and requires differentiation from malignant odontogenic tumors (e.g., ameloblastic carcinoma, clear cell odontogenic carcinoma, and ghost cell odontogenic carcinoma) and intraosseous salivary gland tumors such as mucoepidermoid carcinoma, metastatic tumors, inflammatory lesions, and carcinomas secondarily invading the bone from adjacent structures before confirming the diagnosis. Furthermore, it cannot be distinguished from malignant tumors arising from the oral mucosa if there is evidence of both cortical bone destruction and pathological lesions in adjacent soft tissue, such as ulceration.

Here, we report a case of PIOC that was initially diagnosed as mandibular osteomyelitis secondary to pericoronitis of the impacted left lower third molar. The diagnosis was revised to PIOC based on the histopathological evaluation of granulation tissue surrounding the extraction socket.

## Case presentation

**Figure 1:**
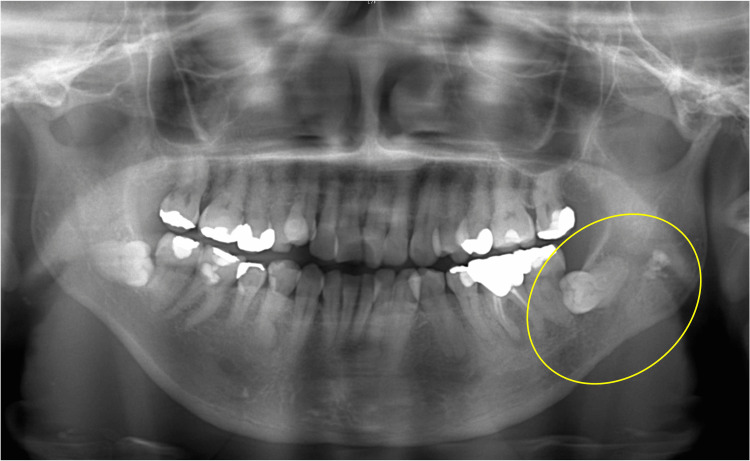
Panoramic radiography at first visit Panoramic radiography showed a slightly radiolucent area in the apical regions of teeth 36 and 37 and no significant findings around tooth 38 (yellow oval).

In March 2017, a 53-year-old woman visited a local dental office with the chief complaint of pain in the left lower third molar. The patient was diagnosed with pericoronitis of the left lower third molar and was referred to our department for tooth extraction. At the initial visit, extraoral findings were normal. Intraoral examination revealed swelling and redness around the left lower molar gingiva, and the left lower third molar (38) was fully impacted. Panoramic radiography showed a slightly radiolucent area in the apical regions of teeth 36 and 37, but no significant findings around tooth 38 (Figure 1).

**Figure 2:**
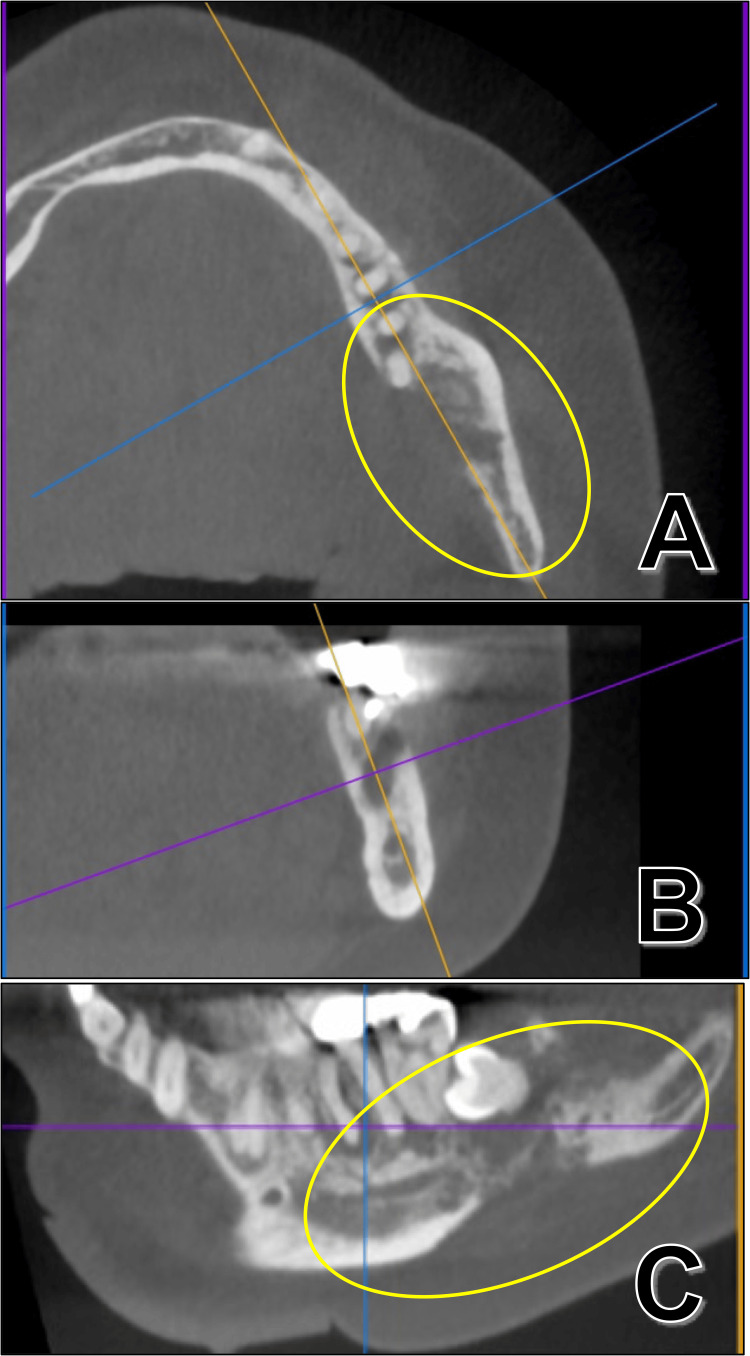
Computed tomography at first visit Axial section (A), coronal section (B), and sagittal section (C) around teeth 36, 37, and 38. Irregular resorption of the lingual cortical bone around teeth 36, 37, and 38 was recognized. Yellow ovals: an important finding due to the destruction of the lingual cortical bone

In contrast, computed tomography (CT) revealed irregular resorption of the lingual cortical bone around teeth 36, 37, and 38 (Figure 2).

**Figure 3:**
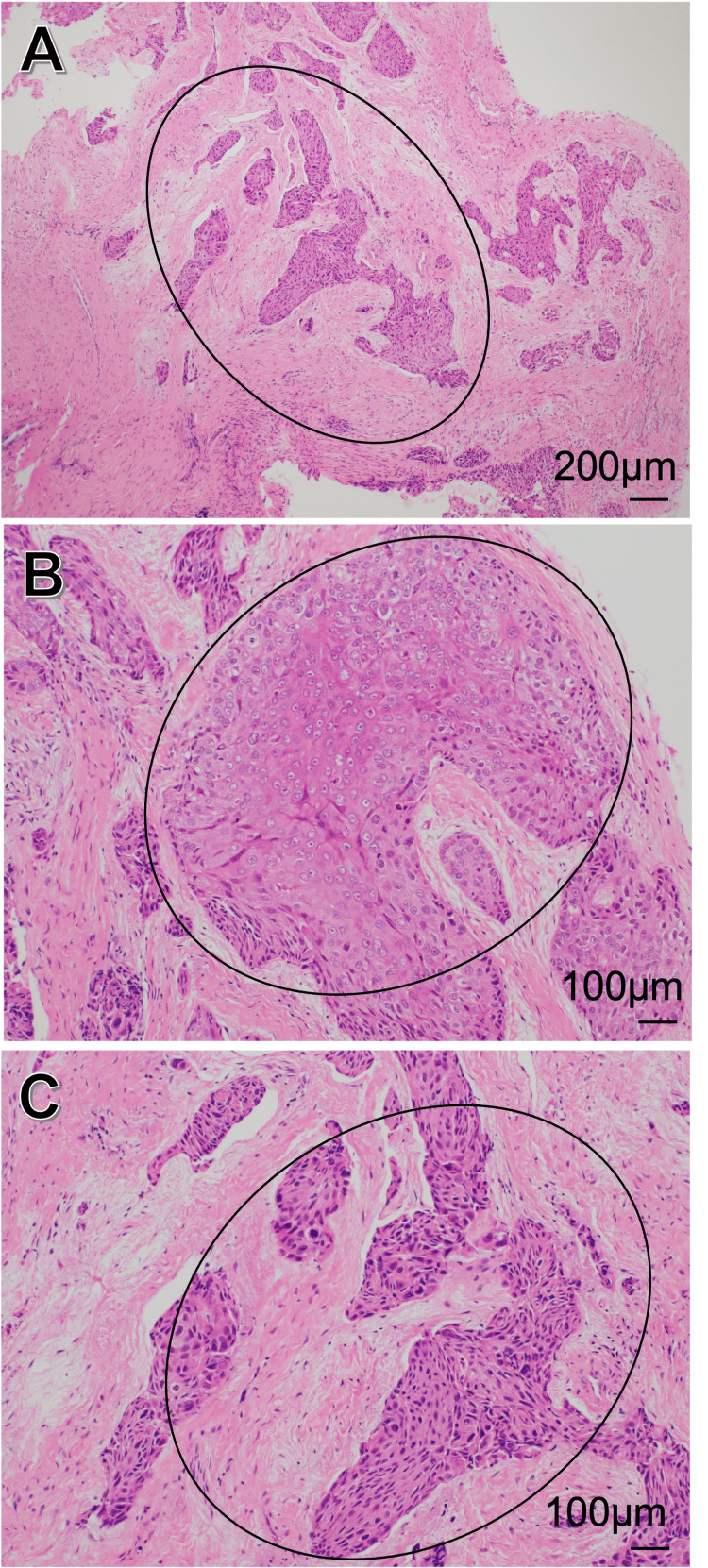
Histological findings of granulation tissues taken from tooth 38 extraction socket Scattering epithelial nests were observed in the granulation tissue (A), which showed keratinization (B) and cytological atypia such as hyperchromatism and atypical mitoses (C). Black ovals: squamous cell carcinoma is observed histopathologically in the granulation tissue of the extraction socket

Based on a clinical diagnosis of mandibular osteomyelitis caused by pericoronitis of tooth 38, tooth extraction was performed under general anesthesia in July 2017. Considering the possibility of malignancy due to the preoperative CT findings of destructive bone resorption, the granulation tissue surrounding the extraction socket was sent for histopathological evaluation. The histopathological diagnosis of the granulation tissue revealed moderately differentiated squamous cell carcinoma. Scattered epithelial nests were observed in the granulation tissue, exhibiting keratinization and cytological atypia such as hyperchromatism and atypical mitoses. Based on these findings, the lesion was diagnosed as squamous cell carcinoma (Figure 3).

**Figure 4:**
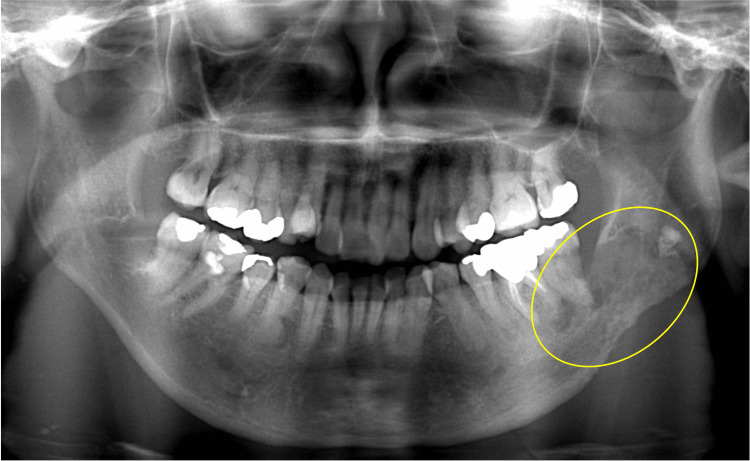
Panoramic radiography after tooth 38 extraction Postoperative examination of the lesion revealed significant bone resorption near tooth 38 and the inferior border of the mandible. Yellow oval: bone destruction has progressed compared to before the tooth extraction, suggesting that it is not a typical case of mandibular osteomyelitis

Postoperative examination of the lesion revealed significant bone resorption near tooth 38 and the inferior border of the mandible on panoramic radiography (Figure 4).

**Figure 5:**
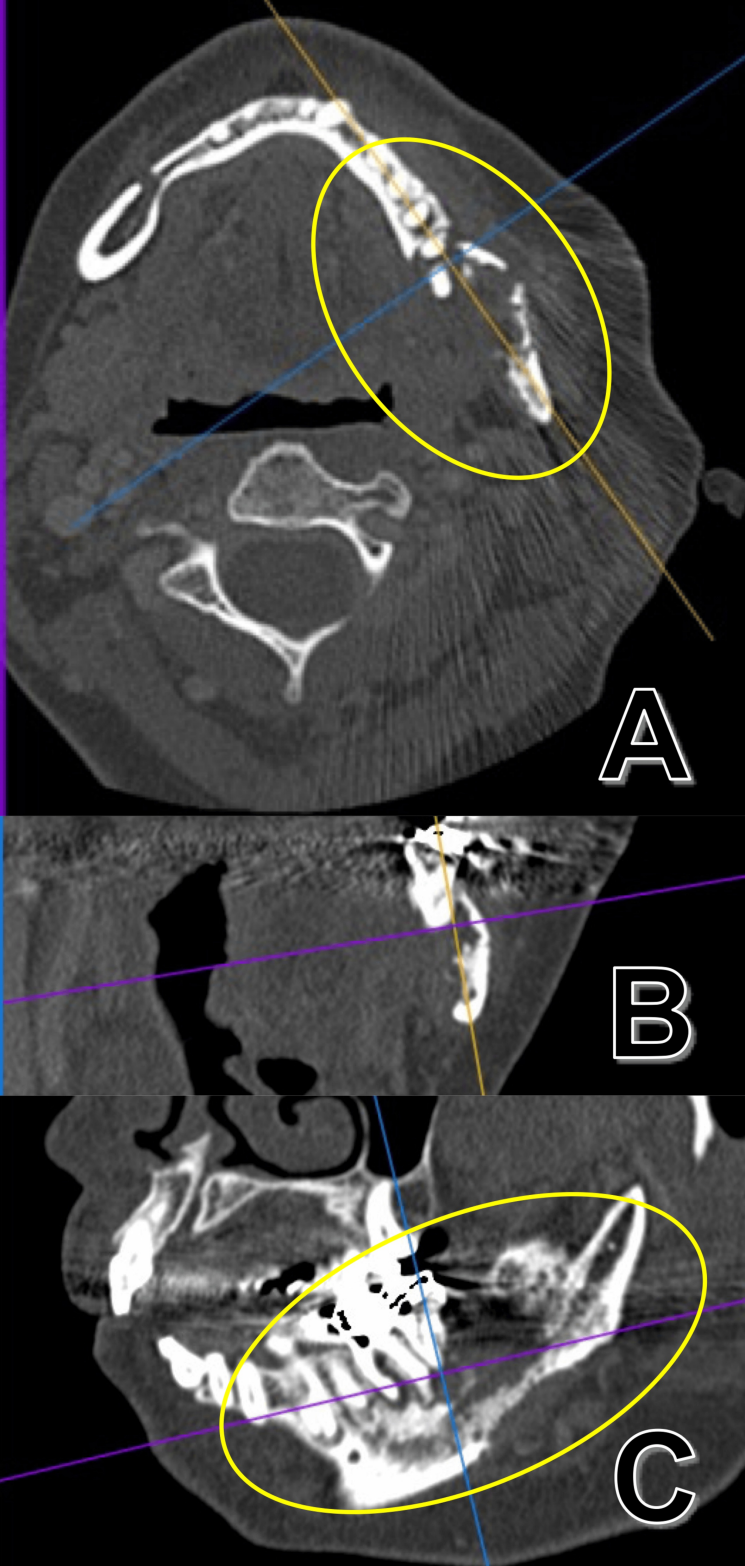
Computed tomography (CT) after tooth 38 extraction (hard tissue mode) Axial sections (A), coronal sections (B), and sagittal sections (C) around the left lower molar area. Irregular destructive resorption of the lingual cortical bone and invasion into the mandibular canal was confirmed. Yellow ovals: the destruction of the lingual cortical bone has progressed compared to the pre-extraction CT images

CT confirmed resorption of the lingual cortical bone and invasion into the mandibular canal (Figure 5).

**Figure 6:**
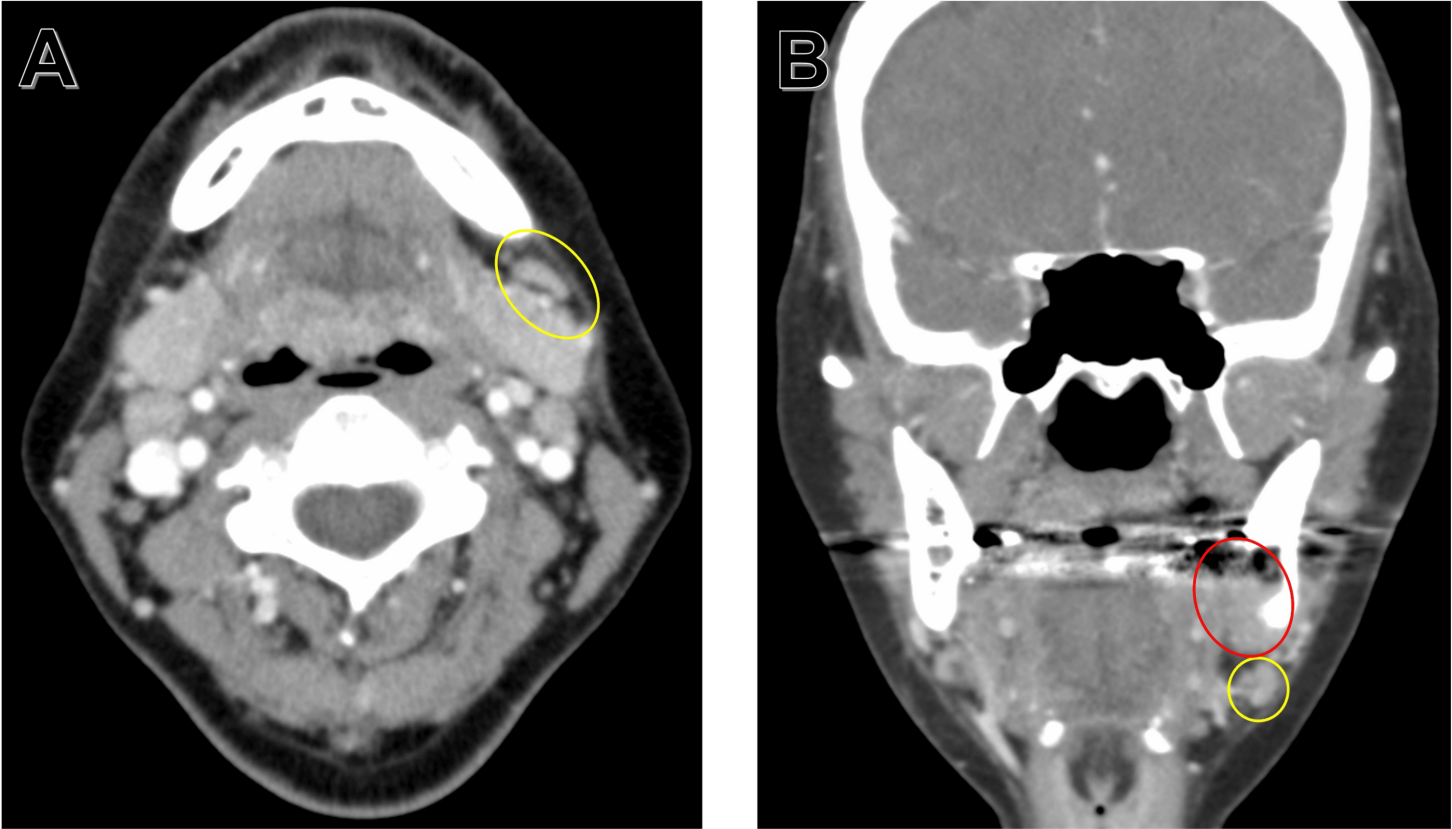
Computed tomography after tooth 38 extraction (soft tissue mode) Coronal section (A) and axial section (B) of the submandibular area. The lesion (red oval) was in close proximity to the left submandibular lymph node (yellow ovals), suggesting the possibility of direct invasion to the lymph node.

In addition, the lesion was in close proximity to the left submandibular lymph node, suggesting the possibility of direct invasion to the lymph node (Figure 6).

**Figure 7:**
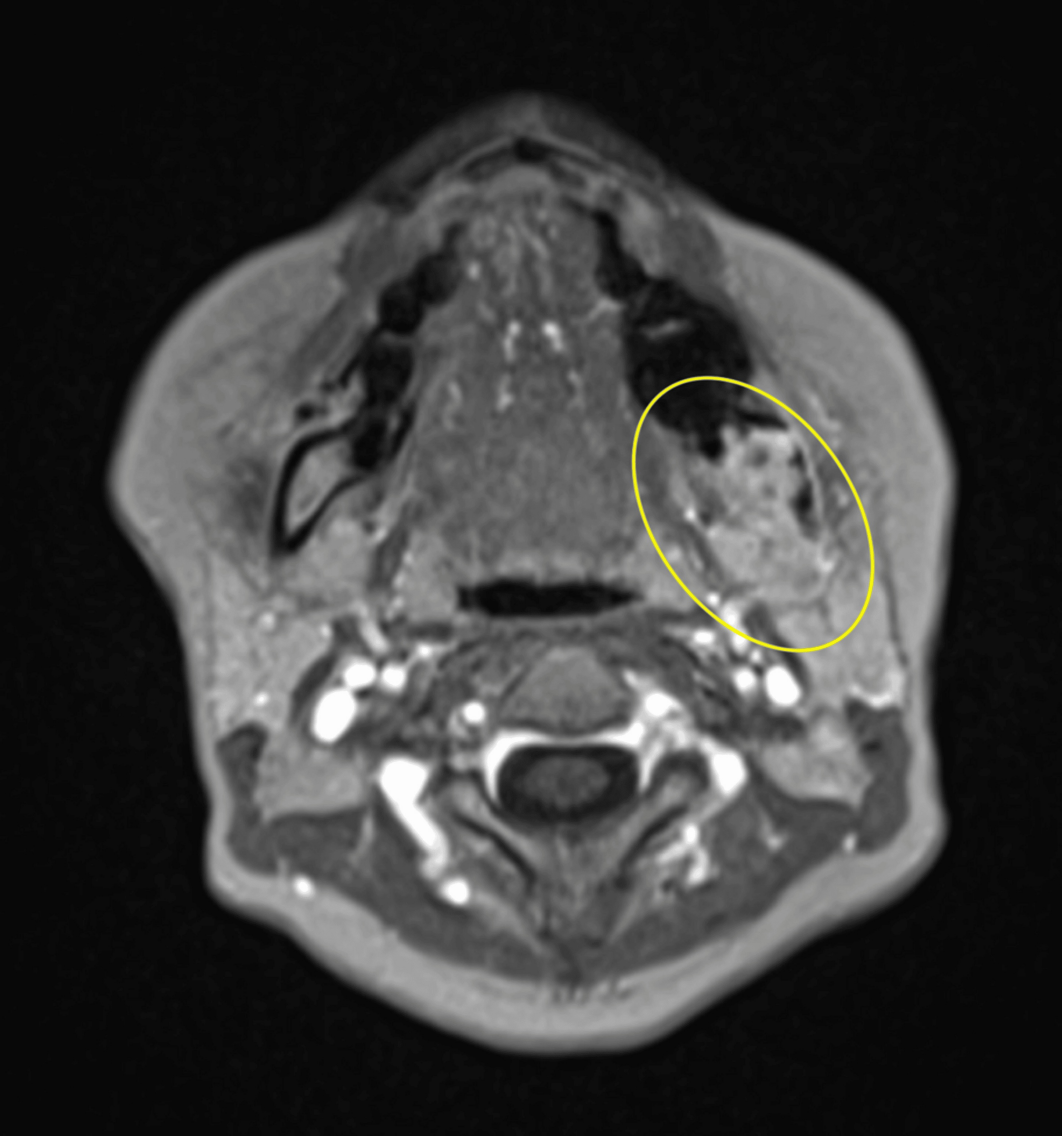
Contrast-enhanced magnetic resonance imaging Contrast-enhanced magnetic resonance imaging demonstrated an early enhancing mass lesion extending from the extraction socket of tooth 38 to the ramus, with invasion into the pterygomandibular space, findings consistent with malignancy. Yellow oval: strongly suggestive of malignancy

Contrast-enhanced magnetic resonance imaging (MRI) demonstrated an early enhancing mass lesion extending from the extraction socket of tooth 38 to the ramus, with invasion into the pterygomandibular space, findings consistent with malignancy (Figure 7).

**Figure 8:**
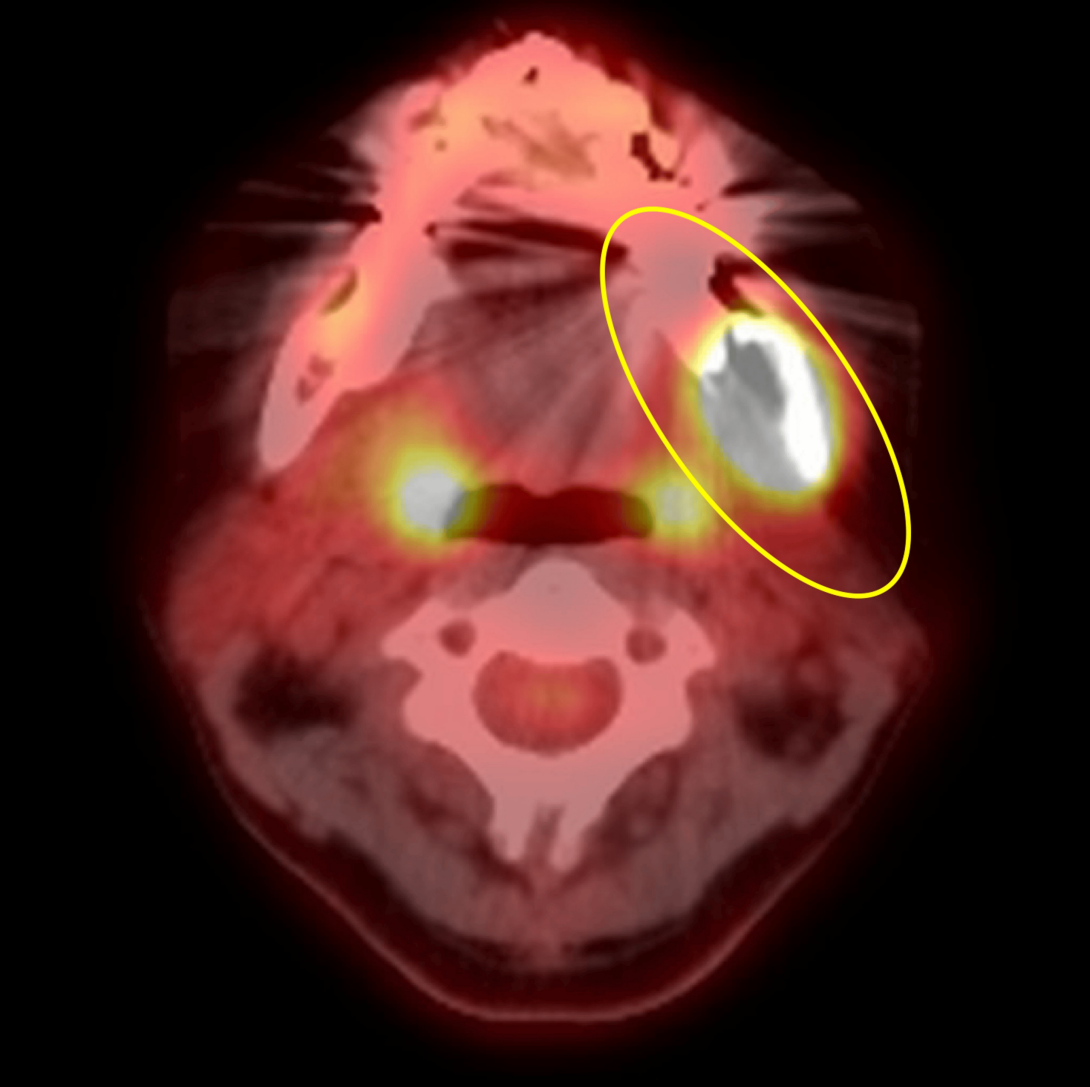
Fludeoxyglucose F18-positron emission tomography after tooth 38 extraction Fludeoxyglucose F18-positron emission tomography; strong accumulation was observed in the mass lesion extending from the extraction socket to the left ramus. Yellow oval: strongly suggestive of malignancy

Ultrasonographic evaluation revealed no evidence of metastases to the cervical lymph nodes. On Fludeoxyglucose F18-positron emission tomography (PET), strong accumulation was observed in the mass lesion extending from the extraction socket to the left ramus (Figure 8). However, no abnormal accumulation suggestive of distant metastasis was observed.

**Figure 9:**
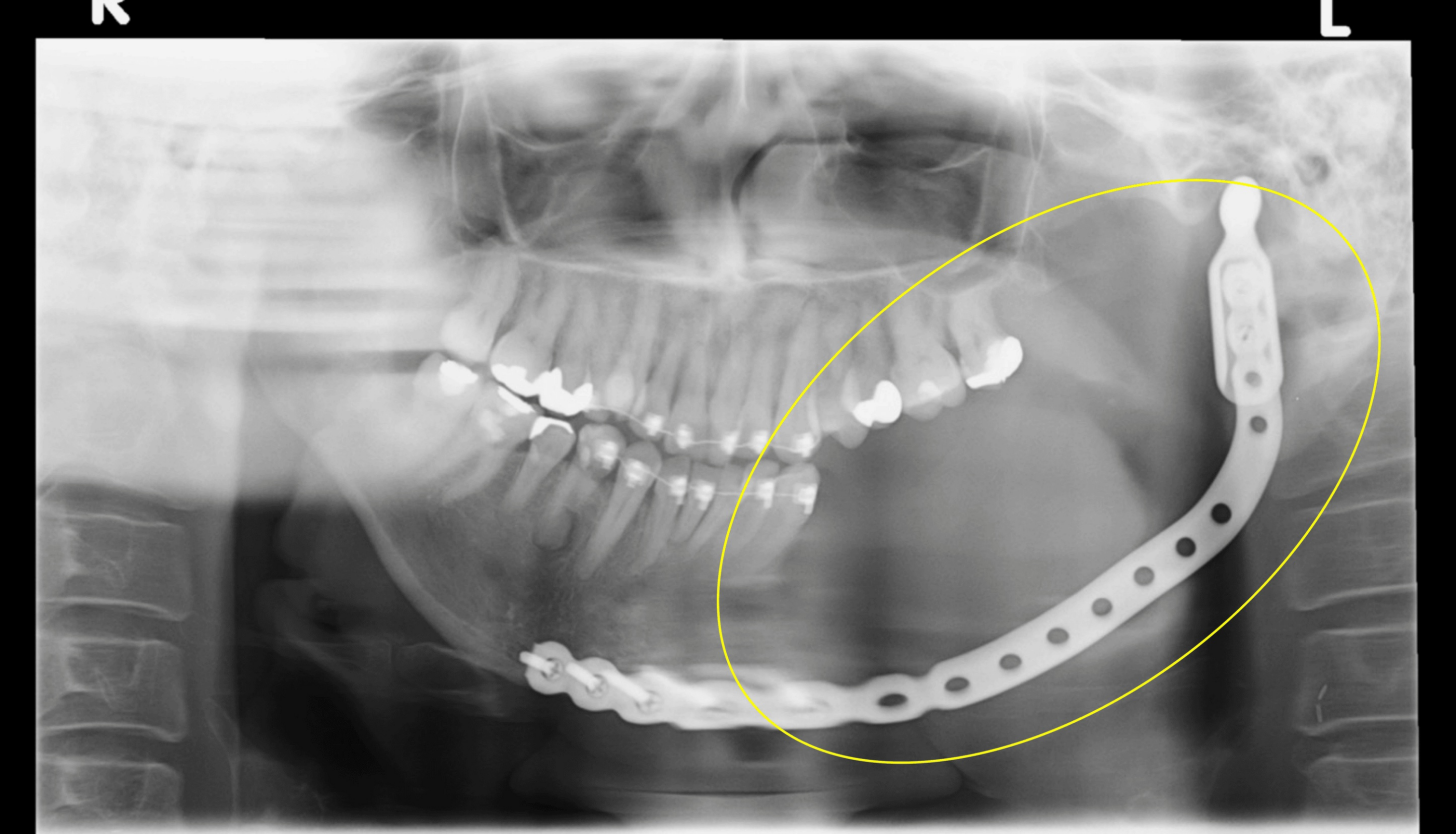
Postoperative panoramic radiography Yellow oval: the pathological area has been appropriately resected

Based on these findings, the patient was diagnosed with PIOC and underwent left-sided neck dissection, left-side hemi-mandibulectomy, and reconstructive surgery using a titanium plate and a rectus abdominis free flap in August 2017 (Figure 9).

**Figure 10:**
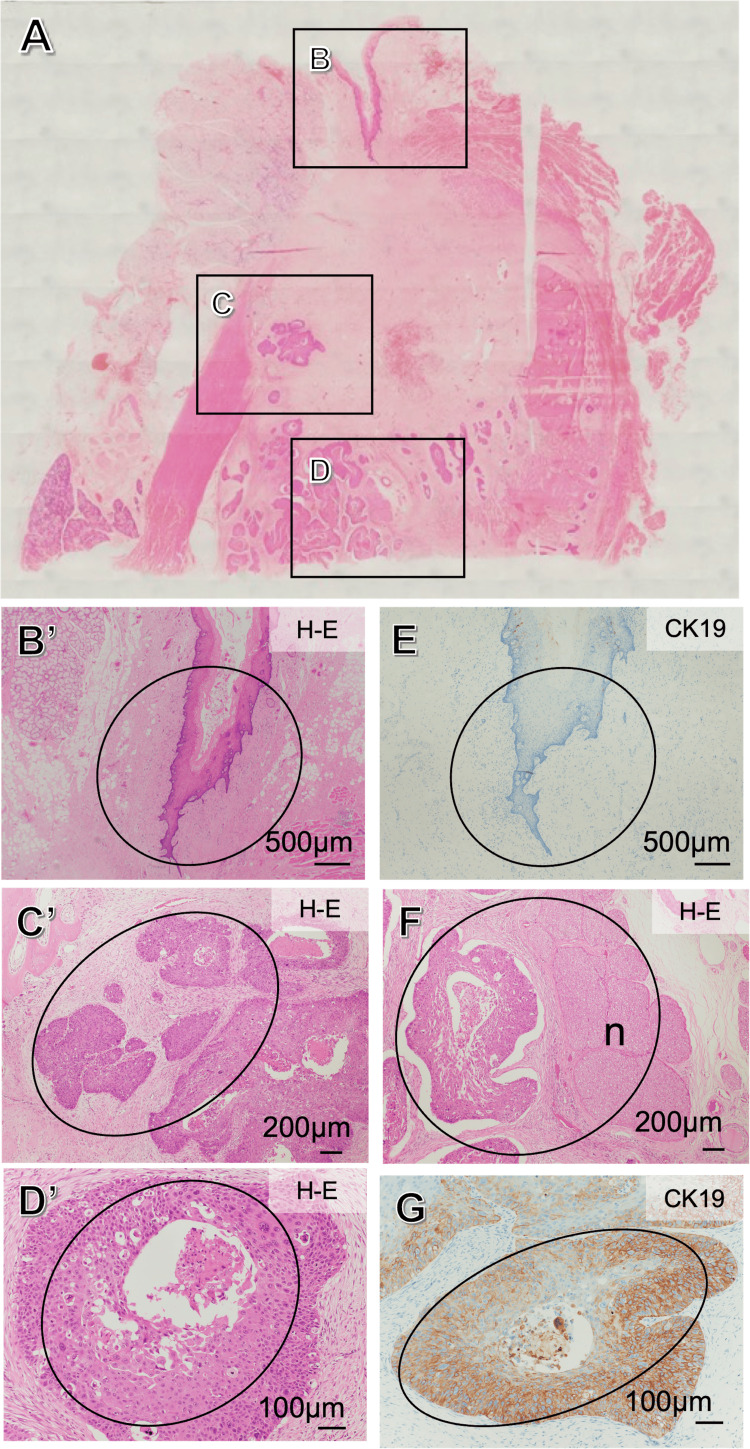
Histological examination of the surgical specimens A representative cross-section of the surgical specimen (A). The intraosseous growth of the cancer nests was confirmed, and that did not show any continuity to the surface epithelium (B-D). The highly magnified images of B, C, and D correspond to panels B', C', and D', respectively. Surface epithelia were negative for CK19 (E), whereas the cancer nest invading the inferior alveolar nerve (n) was recognized (F) and was positive for CK19 (G). Black ovals:  The mucosal epithelium is normal, but squamous cell carcinoma is observed within the jawbone. This finding clearly indicates that it is PIOC PIOC: primary intraosseous carcinoma

Histopathological examination of the surgical specimens confirmed the absence of lymph node metastasis. Histologically, intraosseous growth of cancer nests was observed in the surgical specimens, with no continuity to the surface epithelium. The surface epithelia were negative for CK19, whereas the cancer nests invading the inferior alveolar nerve were positive for CK19, indicating an odontogenic epithelial origin. Thus, the lesion was diagnosed as PIOC, NOS (Figure 10).

Seven years and four months after the surgery, the patient remains in good health, with no evidence of primary recurrence, cervical recurrence, or distant metastasis.

## Discussion

We report a rare instance of PIOC of the mandible, initially diagnosed as mandibular osteomyelitis due to pericoronitis of the left lower third molar, aiming to highlight the diagnostic challenges and indicate the importance of early detection.

PIOC is classified as a rare malignant odontogenic tumor, accounting for 1%-2.5% of all odontogenic carcinomas and approximately 2% of all squamous cell carcinomas arising in the oral cavity [5,6]. The condition most commonly affects individuals in their late 50s, with a male-to-female ratio of 2:1, and is more prevalent in men [7]. As the lesion originates within the jawbone, symptoms often present as tooth pain.

The diagnosis of PIOC is based on the absence of continuity with the oral mucosa, absence of metastatic oral tumors, and presence of squamous cell carcinoma [8]. Radiographic differentiation of PIOC is challenging, as it often appears similar to radicular cysts or other odontogenic cysts [5]. Bodner et al. reported that PIOC may originate from residual cysts (60%), dentigerous cysts (16%), odontogenic keratocysts (14%), or lateral periodontal cysts (1%) [8].

In the present case, histopathological analysis of the granulation tissue from the extraction socket confirmed a diagnosis of squamous cell carcinoma, although the precise origin of the carcinoma cells was unclear. It was hypothesized that the carcinoma arose from the odontogenic residual epithelium proliferated by pericoronitis or from the reduced enamel epithelium surrounding the left lower third molar. Unlike squamous cell carcinomas of oral mucosal origin, PIOC causes destructive resorption of the jawbone, necessitating segmental mandibulectomy or hemi-mandibulectomy as the initial surgical approach [7]. A previous report described a case in which a tumor in the mandibular region exhibited direct invasion into adjacent cervical lymph nodes, suggesting a potential risk of local spread [9]. In light of this finding and the close proximity of the tumor to the cervical lymph nodes in the present case, neck dissection was performed in addition to primary tumor resection to ensure comprehensive disease control and assess possible lymphatic involvement.

A similar case has been documented in which PIOC initially presented with radiographic features resembling a periapical lesion of the lower third molar, leading to tooth extraction. In some instances, the postextraction site was later interpreted as osteomyelitis or delayed healing, which may have contributed to a postponement of the definitive diagnosis and appropriate treatment [10]. In the present case, the lesion initially manifested as mandibular osteomyelitis, which could have led to a similar diagnostic challenge. Given these complexities, a thorough assessment of radiographically suspicious lesions is essential to ensure timely and accurate diagnosis.

To enhance early detection and minimize delays in treatment, the utilization of contrast-enhanced CT and contrast-enhanced MRI should be strongly considered in cases where PIOC is suspected. Additionally, PET-CT, which has demonstrated high efficacy in detecting malignancies with elevated metabolic activity, may provide further insights for comprehensive assessment. These imaging modalities can offer critical information regarding tumor extent, cortical bone involvement, and potential metastasis, thereby facilitating more precise preoperative planning and improving overall patient outcomes.

A standardized treatment protocol for PIOC has not been clearly established in previous systematic reviews [2]. However, it has been reported that the overall survival (OS) of PIOC is comparable to that of stage IV oral squamous cell carcinoma, and treatment strategies similar to those for T3N0 oral cancer may be recommended [11]. Further research is needed to establish a standardized treatment protocol aimed at improving the survival rate of PIOC.

Previous studies have reported that postoperative adjuvant therapy improves survival in univariate analysis. However, in multivariate analysis, it was not identified as an independent prognostic factor, with only positive nodal status, high histological grade, and advanced N classification being significant predictors of survival [12]. In the present case, none of these adverse prognostic factors were present, which justified the decision not to administer postoperative adjuvant therapy. In addition, Li et al. reported a two-year OS rate of 61.3% for PIOC and identified the histopathological absence of metastatic lymph nodes as a key prognostic factor [13]. This may explain the favorable long-term prognosis observed in this case.

Histopathologically, CK19 was evaluated, confirming that the intraosseous lesion originated from odontogenic epithelium, whereas the mucosal epithelium was CK19-negative, supporting a diagnosis of PIOC, NOS. Considering the available tissue samples, a comprehensive immunohistochemical staining panel for all odontogenic malignancies could not be performed. However, the absence of characteristic histological features of ameloblastoma, such as peripheral palisading, reverse polarization, and stellate reticulum-like structures, suggests that ameloblastic carcinoma could be ruled out in this case. In future studies, a more extensive immunohistochemical evaluation incorporating a broader range of markers would be desirable to enhance the diagnostic accuracy of odontogenic malignant tumors and to further elucidate their molecular characteristics.

Currently, due to the limited understanding of its biological behavior, reliable biomarkers for distinguishing PIOC from conventional squamous cell carcinoma have not yet been established [13]. Further research is needed to clarify potential molecular differences and improve diagnostic accuracy. This remains an important area for future investigation.

## Conclusions

In our case, treatment delay was avoided because a biopsy was performed at the time of tooth extraction, considering the possibility of malignancy, and additional surgical treatment was predicted in advance. The prognosis of PIOC is poor, and this may be attributed to delays in diagnosis and treatment, often resulting from the initial misdiagnosis of PIOC as odontogenic cysts, odontogenic tumors, or mandibular osteomyelitis. Given the poor prognosis of PIOC, early diagnosis and timely treatment are crucial to prevent a decline in the patient's quality of life and to improve overall outcomes. General practitioners and oral surgeons, who are often responsible for the initial evaluation, should be aware of the potential for malignancy in radiographically suspicious odontogenic lesions. A thorough radiographic and clinical assessment is essential to ensure appropriate diagnosis and management.
